# Proteomic discovery analysis of quantitatively assessed emphysema in the general population. The MESA Lung Study

**DOI:** 10.1186/s12931-025-03312-8

**Published:** 2025-07-04

**Authors:** Daniel E. Guzman, Lisa Ruvuna, Claire J. Guo, Yifei Sun, Katherine A. Pratte, Ani W. Manichaikul, John S. Kim, Wendy S. Post, Alain G. Bertoni, Norrina B. Allen, Karol E. Watson, James S. Pankow, Eric A. Hoffman, Ruth F. Dubin, Rajat Deo, Igor Z. Barjaktarevic, Eugene R. Bleecker, Christopher B. Cooper, Victor E. Ortega, Annette T. Hastie, Robert Paine, James Michael Wells, Jeffrey L. Curtis, Edwin K. Silverman, Prescott G. Woodruff, Christine Kim Garcia, Jerome I. Rotter, Russell P. Bowler, Peter Ganz, R. Graham Barr

**Affiliations:** 1 Department of Medicine, Columbia University Irving Medical Center, New York, NY USA; 2 Genomic Medicine Institute, Cleveland Clinic, Cleveland, OH USA; 3 Department of Biostatistics and Informatics, Colorado School of Public Health, University of Colorado Anschutz Medical Campus, Aurora, CO USA; 4 National Jewish Health, Denver, CO USA; 5 Center for Public Health, University of Virginia School of Medicine, Charlottesville, VA USA; 6 Department of Medicine, University of Virginia School of Medicine, Charlottesville, VA USA; 7 Department of Medicine, Johns Hopkins University School of Medicine, Baltimore, MD USA; 8 Division of Public Health Sciences, Wake Forest University School of Medicine, Winston-Salem, NC USA; 9 Center for Epidemiology and Population Health, Northwestern University, Chicago, IL USA; 10 Department of Medicine, University of California, Los Angeles David Geffen School of Medicine, Los Angeles, CA USA; 11 Division of Epidemiology and Community Health, School of Public Health, University of Minnesota, Minneapolis, MN USA; 12 Department of Radiology, Carver College of Medicine, University of Iowa, Iowa City, IA USA; 13 Department of Medicine, University of Texas Southwestern, Dallas, TX USA; 14 Department of Medicine, University of Pennsylvania, Philadelphia, PA USA; 15 Department of Medicine, The University of Arizona Health Sciences, Tucson, AZ USA; 16 Department of Medicine, Division of Pulmonary Medicine, Mayo Clinic, Scottsdale, AZ USA; 17 Department of Medicine, Wake Forest School of Medicine, Winston-Salem, MD USA; 18 Division of Respiratory, Critical Care and Occupational Pulmonary Medicine, University of Utah School of Medicine, Salt Lake City, UT USA; 19 Department of Medicine, University of Michigan, and VA Ann Arbor Healthcare System, Ann Arbor, MI USA; 20 Channing Division of Network Medicine and Division of Pulmonary and Critical Care Medicine, Department of Medicine, Brigham and Women’s Hospital, Boston, MA USA; 21 Department of Medicine, University of California San Francisco, San Francisco, CA USA; 22 The Institute for Translational Genomics and Population Sciences, Department of Pediatrics, The Lundquist Institute for Biomedical Innovation at Harbor-UCLA Medical Center, Torrance, CA USA USA; 23 Department of Epidemiology, Columbia University Irving Medical Center, New York, NY USA

**Keywords:** Proteomics, Biomarkers, Emphysema

## Abstract

**Background:**

Pulmonary emphysema occurs frequently in older adults, often without airflow limitation. Its presence predicts symptoms, respiratory hospitalizations and deaths, and all-cause mortality. Proteomics may provide further insights into emphysema pathogenesis and inform therapeutic targets.

**Objective:**

We performed a proteomic discovery analysis of percent emphysema on computed tomography (CT) in a population-based, multiethnic sample from the Multi-Ethnic Study of Atherosclerosis (MESA) Lung Study. Replication was performed in two chronic obstructive pulmonary disease (COPD)-based studies, the SubPopulations and InteRmediate Outcome Measures in COPD Study (SPIROMICS) and the Genetic Epidemiology of COPD (COPDGene) Study.

**Methods:**

MESA recruited participants from the general population in 2000–02. The MESA Lung Study performed full-lung CT scans in 2010–12. Percent emphysema was defined as the percentage of lung voxels < -950 Hounsfield units. Over 7,200 plasma aptamers were measured via SomaScan. Cross-sectional linear and least absolute shrinkage and selection operator (LASSO) regression models were adjusted for demographics, anthropometrics, smoking, renal function, and scanner parameters. Statistical significance was defined as a false discovery rate p-value < 0.05. Gene Ontology (GO)/Reactome enrichment analyses were performed. LASSO-selected proteins’ predictive performance was evaluated.

**Results:**

Among 2,504 participants in the MESA Lung Study, mean age was 69.4 years, 1,291 had ever smoked, and median percent emphysema-like lung was 1.4%. In total, 1,234 aptamers were significantly associated with percent emphysema in the MESA Lung Study, and 35 replicated in the SPIROMICS and COPDGene Studies. Novel associations included protein family with sequence similarity (FAM) 177A1, syntenin-2, ubiquitin carboxyl-terminal hydrolase 25, and uncharacterized protein C20orf173. Previously identified emphysema-associated proteins included soluble advanced glycosylation end product-specific receptor (sRAGE), protein S100-A12, high mobility group protein B1, and roundabout homolog 2. Enrichment analyses identified 40 GO biological processes, including chemokine production and regulation and cell–cell adhesion and regulation, and two Reactome pathways, including RAGE signaling. In tenfold cross-validation, novel proteins were largely retained by LASSO (R^2^ = 5.4%), improved overall model performance (R^2^ = 24.8%), and uniquely explained greater variance in percent emphysema.

**Conclusions:**

This analysis in a general population sample identified novel and previously characterized proteins whose functional roles were validated by GO/Reactome enriched pathways, offering new insights into emphysema pathophysiology and therapeutics.

**Supplementary Information:**

The online version contains supplementary material available at 10.1186/s12931-025-03312-8.

## Introduction

Chronic lower respiratory disease (CLRD) was the third-leading cause of death globally in 2019 [1]. Most mortality from CLRD is from chronic obstructive pulmonary disease (COPD), defined physiologically as the forced expiratory volume in 1 s (FEV_1_) to forced vital capacity (FVC) ratio < 70% [2], and pulmonary emphysema, defined morphologically by destruction of alveolar walls distal to the terminal bronchioles with resultant enlargement of airspaces [3].

Emphysema on autopsy is frequent in the general population [4]. Emphysema assessed quantitatively on computed tomography (CT) as the percentage of emphysema-like lung (hereafter percent emphysema) is also common and only modestly associated with spirometrically-defined COPD [5, 6]. Percent emphysema is associated with activity limitation, impaired lung function, increased respiratory symptoms, hospitalizations, and mortality, and all-cause mortality in the general population independent of airflow limitation and among those without COPD [7–10]. Furthermore, percent emphysema assessed quantitatively on CT, demonstrated to be highly repeatable [11], may be a helpful measure for the discovery of clinically significant circulating biomarkers for emphysema, since it is more specific than spirometric obstruction and acts as a subclinical measure of emphysema.

Understanding of plasma protein associations with emphysema may be helpful for discovering pathways and therapies, particularly in samples not selected on COPD. The only personalized therapy for emphysema is based upon the discovery of a missing protein in serum, repletion of which is disease-modifying. In 1963 Carl-Bertil Laurell and Sten Eriksson noted a missing alpha-1 protein electrophoretic pattern in 5 patients, most of whom had severe emphysema [12]. A subsequent randomized controlled trial of augmentation therapy in patients with alpha-1-antitrypsin deficiency and an FEV_1_ < 80% of predicted demonstrated a slowing of emphysema progression assessed quantitatively on CT [13]. Large-scale examination of the plasma proteome may, therefore, provide mechanistic insights into the pathogenesis of emphysema and ultimately lead to improvements in its prevention and treatment.

Prior observational large-sample proteomic discovery studies in emphysema have been conducted in cohorts that oversampled spirometrically-defined COPD [14–19]. This approach may yield biased estimates of risk for quantitative traits such as percent emphysema that are associated with COPD when sampling fractions are unknown, as is typically the case. Furthermore, most such analyses have been limited to participants with a heavy smoking history, which is an important but far from the only risk factor for emphysema [20]. Identification of proteins that are associated with percent emphysema in a general population sample that does not oversample COPD may, therefore, provide relatively unbiased insights into emphysema pathobiology and risk and may be less confounded by smoking. Moreover, while sampling from a diseased population based on airflow limitation in a cross-sectional analysis may lead to reverse causation, where the disease itself influences plasma protein concentrations, studying proteins in a less diseased population using an early, subclinical measure can more accurately identify proteins involved in the pathogenesis of emphysema. Lastly, prior studies had limited racial/ethnic representation, relatively small sample sizes, or typically examined only a limited repertoire of proteins [14–19].

Thus, we examined associations of plasma proteins with percent emphysema in a population-based multiethnic sample using SomaScan, a proteomic platform that measures thousands of proteins.

## Methods

### Study samples

The Multi-Ethnic Study of Atherosclerosis (MESA) recruited 6,814 participants from six communities who self-identified as White, Black, Hispanic, or Asian race/ethnicity per 2000 US census criteria, were 45–84 years old and free of clinical cardiovascular disease in 2000–02 [21]. The MESA Lung Study randomly enrolled 3,965 MESA participants who consented to genetic analysis, underwent baseline measures of endothelial function, and attended an examination during its recruitment period in 2004–06 with over-sampling of Asians [22]. Of 3,137 participants who underwent full-lung CT scanning from 2010–12 in examination 5, we included all with available plasma proteomic profiles and covariate data irrespective of a prior diagnosis of emphysema or COPD.

Replication was performed in the Subpopulations and Intermediate Outcome Measures in COPD Study (SPIROMICS), a multicenter, longitudinal study that enrolled 2,981 individuals (primarily self-reported White and Black race/ethnicity, as well as Hispanic, American Indian, Alaskan Native, Native Hawaiian, or Other Pacific Islander) 40–80 years old with ≥ 20 packyears of smoking with and without COPD, in addition to non-smoking controls between 2010–15 [23]. Of 2,981 participants with full-lung CT data in visit 1 from 2010–12, we included all with available plasma proteomic profiles and covariate data.

Replication was also performed in the Genetic Epidemiology of COPD (COPDGene) Study, another multicenter, longitudinal study that enrolled 10,652 participants (self-reported non-Hispanic White and Black race/ethnicity) 45–80 years old with ≥ 10 packyears of smoking with and without COPD, in addition to non-smoking controls between 2008–11 [24]. Of 6,120 participants with full-lung CT data in phase two from 2012–17, we included all with available plasma proteomic profiles and covariate data.

### Proteomics

Proteins were quantified using SomaScan (SomaLogic, Inc., Boulder, CO, USA), a high-throughput proteomic platform that utilizes modified aptamers as binding reagents [25, 26]. Bound aptamers were quantified via hybridization microarrays and measured in relative fluorescence units. The platform has excellent specificity and precision, with a median coefficient of variation < 6% [25–29].

SomaScan version 4.1 was used in the MESA Lung Study and SPIROMICS, and version 4.0 in the COPDGene Study. Versions 4.1 and 4.0, respectively, measured 7,289 and 4,979 aptamers mapping to 6,401 and 4,776 unique human proteins that belong to broad biological groups, including receptors, kinases, cytokines, proteases, growth factors, protease inhibitors, hormones, and structural proteins [27, 28].

### Imaging

The MESA Lung Study and SPIROMICS acquired full-lung CT scans following the same protocol [30] with standardized coaching to total lung capacity. Scanner dose was based on body mass index (BMI): 145 mA for 20 kg/m^2^, 180 mA for 20–30 kg/m^2^, and 270 mA for 30 kg/m^2^ [31]. CT scans in the COPDGene Study were acquired using a standardized protocol at total lung capacity with a fixed dose of 200 mA [32]. Scanner manufacturers included General Electric and Siemens across all cohorts, and Philips in the COPDGene Study. Percent emphysema was defined as the percentage of total lung voxels < −950 Hounsfield units (HU) [33]. This threshold was chosen based upon pathology comparisons [34]. The intra-class correlation coefficient (ICC) for percent emphysema on replicate scanning six weeks apart was 0.99 [11].

### Covariates

Height and weight were measured to the nearest 0.1 cm and pound, respectively. BMI was calculated as weight (kg)/height (m)^2^. Age, sex, race/ethnicity, and educational attainment were self-reported, with the latter two variables used as social determinants of health. Educational attainment was defined as no high school degree, high school degree, some college, or college degree. Genetic ancestry was defined using continuous principal components (PCs) [35]. Smoking status was defined as never-, former-, or current cigarette-smoking. In the MESA Lung and COPDGene Studies, never smokers were defined as having smoked < 100 lifetime cigarettes, and in SPIROMICS were defined as having < 1 packyear of cigarette-smoking history. In the MESA Lung and COPDGene Studies, current smoking was defined as a cigarette in the last 30 days, and in SPIROMICS was defined as a cigarette in the last 6 months. Packyears of smoking was calculated as the number of years of smoking × (cigarettes per day/20). Current smoking was confirmed by urinary cotinine level ≥ 100 ng/mL. Estimated glomerular filtration rate (eGFR) was estimated using the four-variable Modification of Diet in Renal Disease equation.

Radiographic interstitial lung abnormalities (ILAs), high attenuation areas (HAAs), and spirometry were measured in the MESA Lung Study, with additional detail available in an online supplement.

### Statistical Analyses

Aptamer levels were natural log transformed to improve normality of distributions. The cross-sectional relationships between percent emphysema (outcome) and aptamers (exposures) were assessed via linear regression models adjusted for height, weight, age, sex, race/ethnicity, PCs of ancestry, educational attainment, smoking status, packyears, urinary cotinine level, eGFR, scanner dose and manufacturer. Beta coefficients were expressed per standard deviation (SD) of each aptamer. Height and weight were adjusted for separately, as percent emphysema varies by each independently [31]. Asian participants in SPIROMICS were grouped with American Indian or Alaska Native, Native Hawaiian or other Pacific Islander, and mixed participants given their small sample size. PCs of ancestry in the COPDGene Study were derived within each subgroup of race/ethnicity and thus not used. Urinary cotinine level was not available in the COPDGene Study. eGFR was available only in the MESA Lung Study and adjusted for as renal function can significantly influence circulating plasma protein concentrations [36]. Statistical significance was defined with a false discovery rate (FDR) p-value (q-value) < 0.05 using the Benjamini–Hochberg method [37] and replication required aptamer coefficient estimates to be in the same direction.

To ensure that replicated aptamers were not confounded by restrictive lung disease or interstitial lung disease (ILD), sensitivity analyses excluding participants with pre-bronchodilator restriction on spirometry and adjusting for ILAs and HAAs, respectively, were conducted. Additional sensitivity analyses used deferent parameterizations of smoking variables, were stratified by smoking status, and used a type III analysis of variance (ANOVA) to evaluate for significant interaction terms by smoking status.

Enrichment analyses were performed separately for Gene Ontology (GO) biological processes and Reactome pathways (including protein interactors) using replicated aptamers [38, 39]. Only pathways that achieved FDR-significance and involved at least three proteins were reported.

A least absolute shrinkage and selection operator (LASSO) regression model was conducted in the MESA Lung Study to address collinearity between replicated aptamers, maintaining clinical covariates as unpenalized predictors. Predictive performance was evaluated using ten-fold cross-validated R^2^ for three models including LASSO-selected aptamers alone, unpenalized covariates alone, and a combined model. The incremental R^2^ for each LASSO-selected aptamer was calculated by adding it individually to the covariate-only model.

Analyses were performed using *RStudio* Version 2024.12.0 + 467 (*R* Foundation, Vienna, Austria), and packages are listed in an online supplement.

## Results

### Characteristics of study samples

**Fig. 1:**
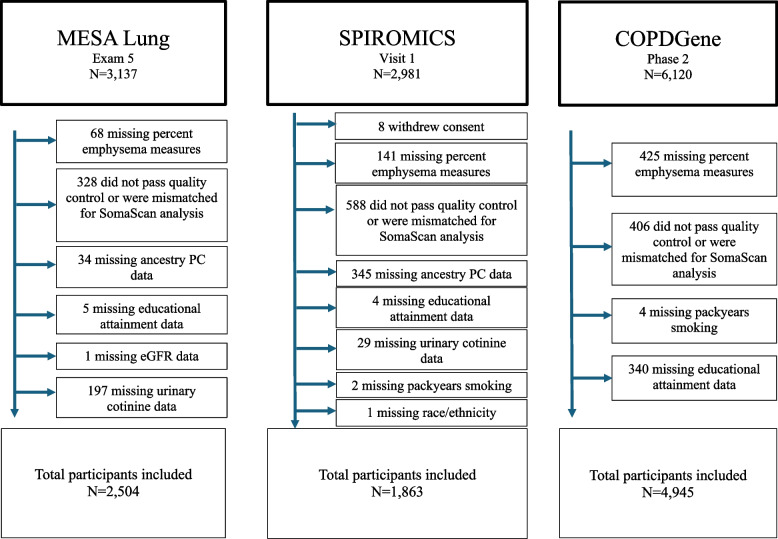
Flow diagram depicting selection of participants included

In the MESA Lung Study examination 5, SPIROMICS visit 1, and second phase of the COPDGene Study, 2,504, 1,863, and 4,945 participants, respectively, had valid measures of percent emphysema, proteomic data, and covariate data (Fig. 1). The baseline characteristics of these participants are described in Table 1. Compared to SPIROMICS and the COPDGene Study, the MESA Lung Study participants consisted of more females; were older; had a greater proportion of Hispanic and Asian, similar proportion of Black, and lower proportion of White participants; and had fewer cumulative packyears, lower exposure to vapors, gas, dust, or fumes, a greater FEV_1_ and FEV_1_/FVC ratio, and lower percent emphysema.

**Table 1:** Characteristics of included participants in the MESA Lung, SPIROMICS, and COPDGene Studies

	**MESA Lung** (*n* = 2,504)	**SPIROMICS** (*n* = 1,863)	**COPDGene** (*n* = 4,945)
Age, mean (SD), years	69.4 (9.3)	63.2 (9.2)	65.3 (8.6)
Male sex, n (%)	1,192 (47.6)	1,012 (54.3)	2,491 (50.4)
Race/ethnicity, n (%)			
White	1,014 (40.5)	1,394 (74.8)	3,521 (71.2)
Black	620 (24.8)	330 (17.7)	1,424 (28.8)
Hispanic	531 (21.2)	74 (4.0)	0 (0)
Asian	339 (13.5)	17 (0.1)	0 (0)
Other	0 (0)	48 (2.6)	0 (0)
Educational attainment, n (%)			
No high school degree	335 (13.4)	208 (11.2)	508 (10.3)
High school degree	449 (17.9)	465 (25.0)	1,143 (23.1)
Some college	729 (29.1)	660 (35.4)	1,371 (27.7)
College degree	991 (39.6)	530 (28.4)	1,923 (38.9)
Height, mean (SD), cm	165.4 ± 9.9	169.9 ± 9.6	169.5 ± 9.7
Weight, mean (SD), kg	78.1 ± 17.5	80.9 ± 17.8	83.6 ± 20.1
BMI less than 20, n (%)	65 (2.6)	109 (5.9)	210 (4.2)
BMI greater than 30, n (%)	836 (33.4)	636 (34.1)	1,888 (38.2)
eGFR, mean (SD), mL/min/1.73m^2^	80.0 (20.2)	NA	NA
Cigarette smoking status, n (%)			
Never	1,213 (48.4)	138 (7.4)	72 (1.5)
Former	1,062 (42.4)	1,043 (56.0)	2,981 (60.3)
Current	229 (9.1)	682 (36.6)	1,892 (38.3)
Packyears of smoking, median (IQR)	12.6 (2—31)	42 (30—60)	40 (27.5—54.7)
Urinary cotinine level, median (IQR), ng/mL	3,205 (1533—6485)	3,250 (1760—6340)	NA
Exposure to Vapors, Gas, Dust, or Fumes, n (%)	552 (22.0)	760 (40.8)	1351 (27.3)
Alpha-1 Antitrypsin Deficiency, n (%)	0 (0)	20 (1.1)	1 (0)
FEV_1_, mean (SD), %	95.3 (19.8)	75.5 (26.4)	78.9 (24.4)
FEV_1_/FVC Ratio, mean (SD)	0.74 (0.1)	0.60 (0.2)	0.68 (0.1)
Scanner manufacturer, n (%)			
General Electric	907 (36.2)	1,080 (58.0)	1,919 (38.8)
Siemens	1,597 (63.8)	783 (42.0)	2,853 (57.7)
Philips	0 (0)	0 (0)	173 (3.5)
Percent emphysema, median (IQR), %	1.4 (0.6—3.1)	2.9 (1.0—10.1)	1.7 (0.5—5.7)
Percent emphysema, mean (SD), %	2.5 (3.2)	7.5 (9.9)	5.6 (9.2)
Traditional Emphysema Subtypes, n (%)			
Centrilobular Emphysema	408 (16.3)	551 (29.6)	2,840 (57.4)
Paraseptal Emphysema	282 (11.3)	522 (28.0)	811 (16.4)
Panlobular Emphysema	64 (2.6)	25 (1.3)	240 (4.9)

Standard deviation reflected as SD and the interquartile range reflected as IQR. Data not available reflected as NA. Percentages may not sum to 100% due to rounding. Body Mass Index (BMI) defined as a BMI. Other races included for SPIROMICS were American Indian or Alaska Native, Native Hawaiian or Other Pacific Islander, and Mixed. Packyears of smoking reflected among ever smokers. Urinary cotinine level measured in current smokers. FEV_1_ and FEV_1_/FVC ratio predicted on spirometry with pre-bronchodilator measures in the MESA Lung Study and post-bronchodilator measures in SPIROMICS and the COPDGene Study

### Proteomics of Percent Emphysema

**Fig. 2:**
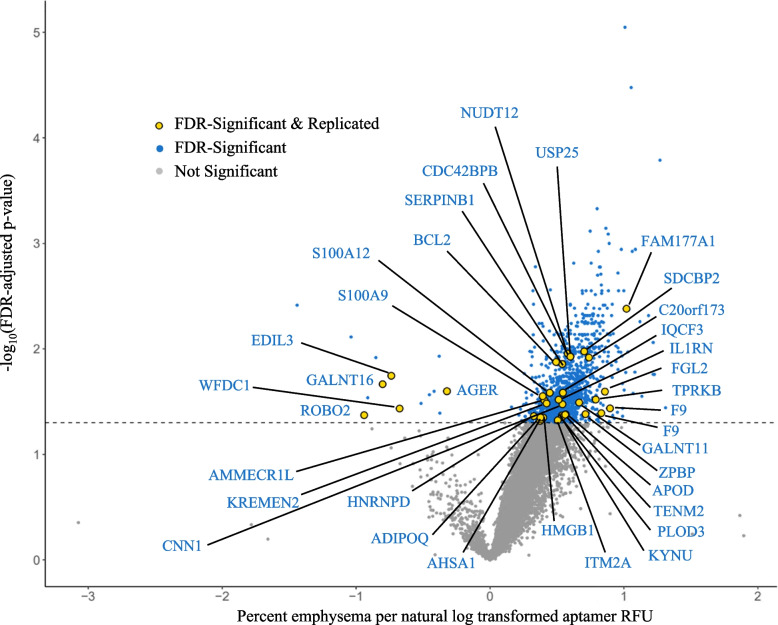
Volcano plot of discovered aptamers in the MESA Lung Study

In the MESA Lung Study, 1,234 aptamers were significantly associated with percent emphysema at a *q*-value < 0.05 after multivariable analysis and are listed with corresponding aptamer and Universal Protein Knowledgebase (UniProt) identifiers in Table E1 and depicted in a volcano plot in Fig. 2. Among these aptamers, 35 replicated in both the SPIROMICS and COPDGene Studies after multivariable analyses and are labeled with corresponding gene identifiers in Table 2 and Fig. 2. The means and SDs of each replicated aptamer are summarized in Table E2. Some of the most significant novel associations included protein family with sequence similarity (FAM) 177A1, syntenin-2, ubiquitin carboxyl-terminal hydrolase 25 (USP25), and uncharacterized protein C20orf173. Some of the most significant proteins with prior associations identified included apoptosis regulator Bcl-2, soluble advanced glycosylation end product-specific receptor (sRAGE), interleukin-1 receptor antagonist protein (IL1RN), roundabout homolog 2 (ROBO2), adiponectin, and high mobility group protein B1 (HMGB1).

**Table 2:** Aptamers associated with percent emphysema discovered in the MESA Lung Study and replicated in the SPIROMICS and COPDGene Studies

		**MESA Lung**		**SPIROMICS**		**COPDGene**	
**Protein Name**	**Gene ID**	***q*-value**	β Coefficient*	***q*-value**	β Coefficient*	***q*-value**	β Coefficient*
Protein FAM177A1	FAM177A1	0.004	4.08	0.049	12.11	0.008	5.86
Syntenin-2	SDCBP2	0.011	2.34	0.046	5.92	< 0.001	4.56
Peroxisomal NADH pyrophosphatase NUDT12	NUDT12	0.011	1.63	0.049	7.16	0.024	3.93
Ubiquitin carboxyl-terminal hydrolase 25	USP25	0.012	1.78	0.042	7.57	0.018	4.40
Serine/threonine-protein kinase MRCK beta	CDC42BPB	0.012	1.74	0.039	9.59	0.005	2.89
Uncharacterized protein C20orf173	C20orf173	0.012	2.68	0.012	21.63	0.003	9.17
Apoptosis Regulator Bcl-2	BCL2	0.013	1.18	0.040	6.58	0.011	2.40
Leukocyte elastase inhibitor	SERPINB1	0.014	1.43	0.008	5.39	0.048	1.37
EGF-like repeat and discoidin I-like domain-containing protein 3	EDIL3	0.018	−2.69	0.002	−5.09	< 0.001	−5.52
Polypeptide N-acetylgalactosaminyltransferase 16	GALNT16	0.022	−3.32	0.002	−8.42	< 0.001	−7.58
Advanced glycosylation end product-specific receptor, soluble	AGER	0.025	−0.52	< 0.001	−5.02	< 0.001	−3.67
Fibroleukin	FGL2	0.025	4.06	0.046	18.00	< 0.001	9.24
Protein S100-A12	S100A12	0.026	1.10	< 0.001	9.52	< 0.001	3.35
IQ domain-containing protein F3	IQCF3	0.026	1.68	0.039	11.93	0.005	8.35
Protein S100-A9	S100A9	0.028	0.87	< 0.001	4.49	< 0.001	1.99
EKC/KEOPS complex subunit TPRKB	TPRKB	0.030	3.62	0.044	21.37	< 0.001	19.72
Interleukin-1 receptor antagonist protein	IL1RN	0.030	1.44	0.049	10.47	0.016	3.64
AMMECR1-like protein	AMMECR1L	0.031	0.97	0.024	5.97	0.036	2.13
Polypeptide N-acetylgalactosaminyltransferase 11	GALNT11	0.032	2.59	0.035	29.38	0.002	15.41
Kremen protein 2	KREMEN2	0.033	1.04	0.049	7.58	0.002	3.64
Calponin-1	CNN1	0.033	1.71	0.042	5.25	< 0.001	5.39
Coagulation factor IX	F9	0.037	4.64	0.041	26.33	< 0.001	15.37
WAP four-disulfide core domain protein 1	WFDC1	0.037	−2.32	0.002	−12.15	< 0.001	−8.08
Coagulation Factor IXab	F9	0.041	4.10	0.049	11.86	0.006	9.77
Zona pellucida-binding protein 1	ZPBP	0.041	3.16	0.033	44.89	< 0.001	34.73
Teneurin-2	TENM2	0.042	2.02	0.045	29.84	0.005	11.60
Apolipoprotein D	APOD	0.042	1.97	0.047	8.29	< 0.001	6.54
Roundabout homolog 2	ROBO2	0.042	−5.33	< 0.001	−41.87	< 0.001	−32.95
Procollagen-lysine,2-oxoglutarate 5-dioxygenase 3	PLOD3	0.043	1.80	0.002	13.62	< 0.001	5.25
Heterogeneous nuclear ribonucleoprotein D0	HNRNPD	0.043	0.67	0.044	4.19	< 0.001	2.92
Kynureninase	KYNU	0.044	1.82	0.048	15.74	0.031	5.75
Adiponectin	ADIPOQ	0.045	0.80	0.030	3.63	< 0.001	2.82
High mobility group protein B1	HMGB1	0.045	1.00	0.025	8.29	< 0.001	5.64
Integral membrane protein 2A	ITM2A	0.047	1.66	0.049	19.10	0.006	11.34
Activator of 90 kDa heat shock protein	AHSA1	0.048	0.90	0.028	6.24	0.037	2.09

^*^Multivariable difference in percent emphysema per natural log-scale standard deviation (SD) of each aptamer adjusted for age, height, weight, sex, race/ethnicity, PCs of ancestry, educational attainment, smoking status, packyears, urinary cotinine level, eGFR, and CT scanner dose and manufacturer

Exclusion of individuals with evidence of restriction on spirometry in the MESA Lung Study and adjustment for ILAs and HAAs yielded similar results with 31–33 of 35 replicated aptamers remaining FDR-significant (Table E3). Alternative adjustment for smoking status also yielded similar results with 32 of 35 replicated aptamers remaining FDR-significant (Table E4).

Stratification by smoking status in the original multivariable model resulted in 16, 26, and 5 aptamers with statistically significant 95% confidence intervals for current-, former-, and never-smokers, respectively (Figure E1). After ANOVA, only 8 had statistically significant interaction terms with smoking status (Figure E1).

### Pathway Analyses

**Fig. 3:**
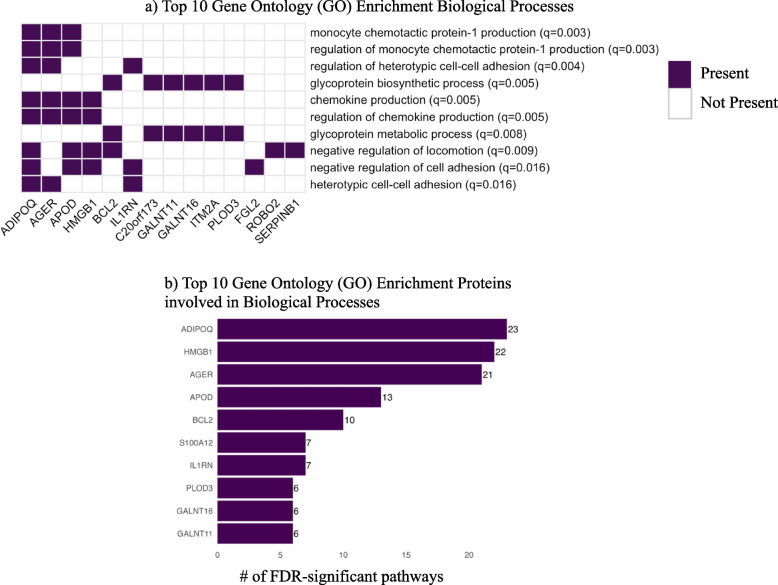
**a** and **b **Gene Ontology (GO) Biological Processes for replicated aptamers **a** and **b**

GO pathway enrichment analysis of replicated aptamers identified 40 biological processes (Table E5). The top 10 most significant were depicted in Fig. 3a and included monocyte chemotactic protein-1 production and regulation, heterotypic cell–cell adhesion and regulation, glycoprotein biosynthetic and metabolic processes, chemokine production and regulation, and negative regulation of locomotion and cell adhesion. The top 10 proteins most represented in significant biological processes are depicted in Fig. 3b and included adiponectin, HMGB1, sRAGE, apolipoprotein D, Bcl-2, protein S100-A12, IL1RN, procollagen-lysine,2-oxoglutarate 5-dioxygenase 3 (PLOD3), and polypeptide N-acetylgalactosaminyltransferases 11 and 16 (GALNT11 and GALNT16).

Reactome pathway analysis demonstrated overrepresentation of three proteins, sRAGE, HMGB1, and protein S100-A12, involved in RAGE signaling and tumor necrosis factor receptor-associated factor 6 (TRAF6) mediated nuclear factor-kappa-light-chain-enhancer of activated B cells (NF-kB) activation (Table 3).

**Table 3:** Reactome pathway analysis demonstrating overrepresentation of proteins associated with percent emphysema

Pathway name	Number of proteins found in pathway	Protein names found in pathway	q-value
Advanced glycosylation endproduct receptor signaling	3	Soluble advanced glycosylation end product-specific receptor, high mobility group protein B1, and protein S100-A12	0.015
TRAF6 mediated NF-kB activation	3	Soluble advanced glycosylation end product-specific receptor, high mobility group protein B1, and protein S100-A12	0.015

### LASSO regression and predictive performance

**Fig. 4:**
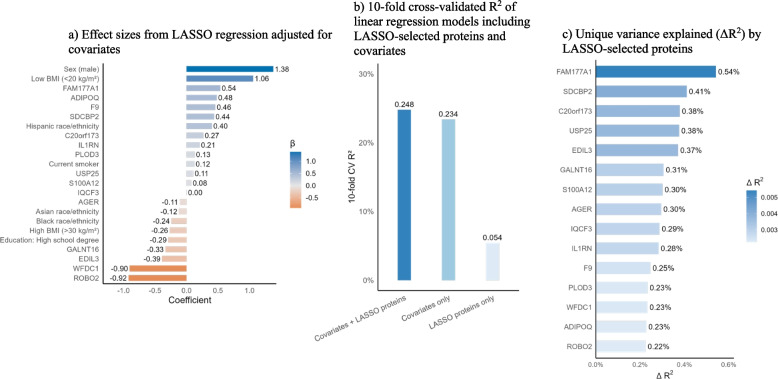
LASSO regression and predictive performance **a**, **b**, and **c**

LASSO regression selected 15 aptamers most strongly associated with percent emphysema, depicted in Fig. 4a. Of these, protein FAM177A1 and adiponectin demonstrated the strongest positive associations with percent emphysema, while ROBO2 and WAP four-disulfide core domain protein 1 (WFDC1) demonstrated the strongest inverse associations. Of the covariates, male sex and low BMI had the strongest positive associations with percent emphysema. In a ten-fold cross-validation, the LASSO-selected aptamers alone explained 5.4% of the variance, the covariates alone explained 23.4% of the variance, and the combined model explained 24.8% of the variance in percent emphysema (Fig. 4b). Lastly, the unique added variance of each LASSO-selected aptamer when added to the covariate only model is depicted in Fig. 4c. Notably, protein FAM177A1, syntenin-2, and uncharacterized protein C20orf173 explained an additional 0.54%, 0.41%, and 0.38% of the variance in percent emphysema, respectively.

## Discussion

This is the first study of which we are aware to utilize high-throughput relatively agnostic proteomic profiling to identify proteins associated with percent emphysema, a subclinical quantitative measure of emphysema, on CT independent of lung function in a multi-ethnic population-based cohort. We report several novel associations of proteins with percent emphysema (e.g., protein FAM177A1, syntenin-2, USP25, and uncharacterized protein C20orf173) as well as known ones (e.g., apoptosis regulator Bcl-2, sRAGE, IL1RN, ROBO2, adiponectin, and HMGB1) that together may provide new insights into disease pathophysiology and drug targets. GO and Reactome enrichment analyses implicated several inflammatory- and cell-signaling-related pathways, amongst others, suggesting multiple pathogenic processes and mechanistic pathways can occur early in the disease course. A LASSO regression model adjusting for covariates selected 15 proteins most associated with and predictive of percent emphysema, emphasizing the need for multiple biomarkers to predict disease [40]. Most associations remained FDR-significant when adjusting for measures of restrictive lung disease and ILD, improving the specificity of these proteins for emphysema. While stratification by smoking status resulted in some effect size differences across strata, few interactions were statistically significant, suggesting that most protein associations with percent emphysema were consistent across smoking groups.

The discovery of several novel proteins associated with percent emphysema highlights an important advantage of discovery-based proteomic analyses in identifying associations not previously recognized. Individually, many of these proteins have roles in inflammatory signaling, including protein FAM177A1 (inhibitor of IL1 beta-induced signaling) [41], fibroleukin (may play a role in mucosal lymphocyte function) [42], and USP25 (deubiquitinating enzyme that modulates the Wnt/beta-catenin pathway) [43, 44]; endothelial regulation, including EGF-like repeat and discoidin I-like domain-containing protein 3 (promotes angiogenesis) [45, 46] and coagulation factors IX and IXab (vitamin K-dependent proteins involved in clotting and regulation of endothelial permeability) [47]; cellular reorganization/movement, including serine/threonine-protein kinase myotonic dystrophy protein kinase-related cell division cycle 42-binding kinase (MRCK) beta (regulates cytoskeleton reorganization and cellular migration) [48] and GALNT11 (catalyzes glycosylation and modulates cilia) [49]; and extracellular matrix modification, including PLOD3 (enzyme involved in type IV collagen modification and probable basement membrane formation) [50], amongst others. Together, many of these proteins are involved in GO enrichment biological processes, including three of the top ten most significant processes identified in this analysis: glycoprotein biosynthetic and metabolic processes, and negative regulation of cell adhesion. The cellular mechanisms carried out by these proteins align with previously described components of emphysema pathophysiology, including inflammation, alveolar epithelial and endothelial alterations, and structural remodeling [3, 51, 52].

In addition to these novel discovered proteins, several proteins identified in this study have been described in prior literature related to emphysema pathogenesis. The most notable is sRAGE, a soluble form of the membrane receptor known for its activation of NF-kB [53]. sRAGE has consistently been found to be inversely associated with the presence and severity of emphysema in individuals with airflow limitation [14, 16, 54]. The replication of this finding with percent emphysema in this general population sample suggests that this association may be independent of airflow limitation. Additionally, prior studies have hypothesized that other RAGE proteins may play a role in emphysema pathogenesis [55, 56], including HMGB1 (a transcription enhancer, cytokine, and inflammation promoter) [57] and protein S100-A12 (an alarmin released by activated neutrophils and monocytes in inflammatory responses) [58]; our GO and Reactome pathway enrichment results support their involvement. Other notable proteins previously described include apoptosis regulator Bcl-2 (an apoptosis suppressor downregulated in COPD resulting in emphysema progression) [59, 60], IL1RN (an inflammatory antagonist decreased in emphysema, contrary to our finding of a positive association) [61, 62], ROBO2 (a receptor downregulated and inversely associated with progression of COPD and involved in alveolar regeneration in a murine model of cigarette-smoke induced emphysema) [63, 64], and adiponectin (mediated inflammation in a murine model of cigarette smoke-induced emphysema) [65].

After LASSO regression, several novel proteins were selected as having the strongest covariate-adjusted associations with percent emphysema and the largest incremental variance contributions in percent emphysema, namely WFDC1, protein FAM177A1, coagulation factor IX, syntenin 2, and uncharacterized protein C20orf173. In contrast, LASSO-selected proteins previously implicated in emphysema, namely adiponectin, IL1RN, S100-A12, sRAGE, and ROBO2, showed smaller relative coefficients (except for ROBO2 and adiponectin) and added less unique variance in percent emphysema, suggesting overlap with other clinical predictors of percent emphysema or lower independent effects. Overall, inclusion of these selected proteins in a combined model with covariates modestly improved cross-validated R^2^, suggesting that while traditional clinical predictors explain most of the variation in percent emphysema, these proteins may provide further insight into underlying emphysema pathophysiology.

Alpha-1 antitrypsin was not found in this proteomic analysis of percent emphysema likely since the present analysis is suited to detect common, rather than rare, variation of plasma protein concentrations in a general population sample. Variants of SERPINA1 generally do not attain statistical significance even in very large genome-wide analyses of lung function [66] making it unlikely that alpha-1 antitrypsin would be significant in this analysis. Likewise, the absence of certain COPD- or smoking-related proteins, such as matrix metalloelastase (MMP12), may be a consequence of discovering proteins in a general population sample with relatively normal spirometry and few current smokers [67, 68].

A strength of this study is its large sample size obtained from three observational cohorts, providing greater statistical power compared to independently analyzed observational studies. Furthermore, the discovery cohort in this study was population-based and not selected for the presence of lung disease, thus reducing potential selection bias. This study design is significant because it allows for the identification of proteins in a broader population with lower percent emphysema, which were then successfully replicated in studies with higher emphysema prevalence. A recently published proteomic analysis using a similar study design of discovery in a general population cohort led to the creation of a risk score of accelerated lung function decline that was applicable in the COPDGene Study using 32 overlapping FDR-significant plasma proteins [69]. However, these proteins did not overlap with replicated proteins found in our study, likely given different endpoint measures, which partially emphasizes the differences in disease biology that underly emphysema and COPD. Our approach may have limited the number of replicated aptamers due to clinical and disease heterogeneity across cohorts, as we did not have a general population cohort available to replicate our findings. Moreover, the SPIROMICS and COPDGene Studies did not have eGFR available to adjust for and could have affected replication results [36]. Additionally, the cross-sectional design of this study limits the ability to determine whether protein concentration changes occurred prior to or after the development of emphysema, though measuring a subclinical endpoint such as percent emphysema in a general population cohort makes it less likely that proteins discovered were a result of disease. Nevertheless, this observational study cannot prove that the replicated proteins cause emphysema. Lastly, given SomaScan is an exploratory discovery platform, identified biomarkers should undergo technical validation, to confirm aptamer specificity, and additional clinical validation.

In summary, we have discovered 1,234 proteins associated with percent emphysema in a general population cohort, of which 35 remained FDR-significant after replication in two additional cohorts. Associations included both novel and previously described emphysema-associated proteins whose functional roles were validated by GO and Reactome enriched pathways. Fifteen LASSO-selected proteins were found to modestly predict percent emphysema and largely made up of novel proteins that uniquely explained greater variance in percent emphysema. Additional multi-omic investigations into these proteins and their associated pathways should be pursued to help elucidate new pathophysiologic mechanisms of emphysema.

## Supplementary Information

Supplementary Material 1.

## Data Availability

Data was obtained through the National Institutes of Health (NIH) and National Heart, Lung, and Blood Institute (NHLBI) funded cohorts the Multi-Ethnic Study of Atherosclerosis (MESA) Lung Study, the SubPopulations and InteRmediate Outcome Measures Study (SPIROMCS), and the Genetic Epidemiology of COPD (COPDGene) Study. Due to privacy and ethical restrictions, these data are not publicly available and can be accessed through controlled-access repositories upon request.
