# Supplementary material for: Proteomic discovery analysis of quantitatively assessed emphysema in the general population. The MESA Lung Study
Source: Respir Res. 2025 Jul 4;26:236. doi: 10.1186/s12931-025-03312-8 (PMC12228189; doi:10.1186/s12931-025-03312-8)
Supplement: Supplementary file 1 — Supplementary Material 1. [file 12931_2025_3312_MOESM1_ESM.docx]

**Proteomic discovery analysis of quantitatively assessed emphysema in the general population. The MESA Lung Study**

Daniel E. Guzman, Lisa Ruvuna, Claire J. Guo, Yifei Sun, Katherine A. Pratte, Ani W. Manichaikul, John S. Kim, Wendy S. Post, Alain G. Bertoni, Norrina B. Allen, Karol E. Watson, James S. Pankow, Eric A. Hoffman, Ruth F. Dubin, Rajat Deo, Igor Z. Barjaktarevic, Eugene R. Bleecker, Christopher B. Cooper, Victor E. Ortega, Annette T. Hastie, Robert Paine III, James Michael Wells, Jeffrey L. Curtis, Edwin K. Silverman, Prescott G. Woodruff, Christine Kim Garcia, Jerome I. Rotter, Russell P. Bowler, Peter Ganz, R. Graham Barr

**Online Data Supplement**

**Supplemental Methods**

**Study Samples**

The Multi-Ethnic Study of Atherosclerosis (MESA) recruited 6,814 participants from six communities who self-identified as White, Black, Hispanic, or Asian race/ethnicity per 2000 US census criteria, were ages 45-84 years old and free of clinical cardiovascular disease in 2000-02 (E1). The MESA Lung Study enrolled 3,965 MESA participants who were sampled randomly among those who consented to genetic analysis, underwent baseline measures of endothelial function, and attended an examination during its recruitment period in 2004-06 (99%, 89%, and 91% of the MESA cohort, respectively) with over-sampling of Asians (E2). Of 3,137 participants who underwent full-lung CT scanning from 2010-12 in Exam 5, we included all with available plasma proteomic profiles and covariate data irrespective of a prior diagnosis of emphysema or COPD.

Replication was performed in the Subpopulations and Intermediate Outcome Measures in COPD Study (SPIROMICS), a multicenter longitudinal COPD case-control study that enrolled 2,981 individuals (primarily self-reported White and Black race/ethnicity, as well as Hispanic, American Indian, Alaskan Native, Native Hawaiian, or Other Pacific Islander) 40-80 years old with ≥20 tobacco smoking packyears with and without COPD, in addition to non-smoking controls between 2010-15 (E3). Of 2,981 participants with full-lung CT data in Visit 1 from 2010-12, we included all with available plasma proteomic profiles and covariate data.

Replication was also performed in the Genetic Epidemiology of COPD (COPDGene) Study, a multicenter longitudinal COPD case-control study that enrolled 10,652 participants (self-reported non-Hispanic White and Black race/ethnicity) 45-80 years old with ≥10 tobacco smoking packyears with and without COPD, in addition to non-smoking controls between 2008-11 (E4). Of 6,120 participants with full-lung CT data in phase two from 2012-17, we included all with available plasma proteomic profiles and covariate data.

The studies were approved by local Institutional Review Boards and the National Heart Lung Blood Institute (NHLBI), and involved participants gave informed written consent.

**Proteomics**

In the MESA Lung Study, 0.5mL EDTA plasma samples were collected in fasting participants via phlebotomy, processed within 30 minutes, and frozen at -80°C. Requested sample volumes for analysis were thawed, pipetted into a new vial, and shipped on dry ice. In SPIROMICS and the COPDGene Study, sample collection differed by volume, 150µL and 250µL respectively, and processing, completed the same day with varying times. COPDGene samples were collected in non-fasting participants.

Proteins were quantified using SomaScan (SomaLogic, Inc., Boulder, CO, USA), a high-throughput proteomic platform that utilizes modified aptamers as binding reagents in a sample volume of 55µL, as previously described (E5-E6). Bound aptamers were quantified via DNA amplification on a microarray with a readout in relative fluorescence units. The platform has excellent specificity and precision, with a median coefficient of variation <6% (E5-E9). Adaptative normalization by maximum likelihood (ANML), which normalizes platform measurements to a healthy population reference, was used in all three cohorts. Quality control of the platform has been previously described (E8-E9) and is additionally available on the SomaLogic website.

SomaScan version 4.1 was used in the MESA Lung Study and SPIROMICS, and version 4.0 in the COPDGene Study. Versions 4.1 and 4.0, respectively, measured 7,288 and 4,979 aptamers mapping to 6,401 and 4,776 unique human proteins that belong to broad biological groups, including receptors, kinases, cytokines, proteases, growth factors, protease inhibitors, hormones, and structural proteins (E7-E8).

**Imaging**

The MESA Lung Study and SPIROMICS acquired full-lung CT scans at suspended full inspiration following the same protocol (0.625-0.75mm slice thickness, 0.984-1.0 pitch, 0.5s rotation time, 120kVp) (E10) with standardized coaching to total lung capacity. The milliampere level (dose) in these two cohorts was based on body mass index (BMI): 145mA for 20kg/m^2^, 180mA for 20–30kg/m^2^, and 270mA for 30kg/m^2^; images were reconstructed at 0.625mm (E11). CT scans in the COPDGene Study were acquired using a standardized protocol at total lung capacity using a dose of 200mA for all subjects (E12). Scanner calibration was confirmed throughout the studies with monthly lung phantom measures. Scanner manufacturers included General Electric and Siemens across all three cohorts, as well as Philips in the COPDGene Study.

Percent emphysema was defined as the percentage of total lung voxels <-950 Hounsfield units (HU) (E13). This threshold was chosen based upon pathology comparisons (E14). The intra-class correlation coefficient (ICC) for percent emphysema on replicate scanning six weeks apart following this protocol was 0.99 (E15).

**Clinical Covariates**

Height and weight were measured to the nearest 0.1 cm and pound, respectively. BMI was calculated as weight (kg)/height (m)^2^. **Age, sex, race/ethnicity, and educational attainment were self-reported**, with the latter two variables used as social determinants of health. Educational attainment was defined as no high school degree, high school degree, some college, or college degree. Genetic ancestry was defined using continuous principal components (PCs) (E16). The first three PCs account for 86% of the total observed variation of regions of geographic ancestry in the MESA Lung Study with the relative value of additional PCs beyond the third being very small (E16). We, therefore, utilized PCs of ancestry 1-3.

Smoking status was defined as never-, former-, or current cigarette-smoking. In the MESA Lung and COPDGene Studies, never smokers were defined as having smoked <100 lifetime cigarettes, and in SPIROMICS were defined as having <1 packyear of cigarette-smoking history. In the MESA Lung and COPDGene Studies, current smoking was defined as a cigarette in the last 30 days, and in SPIROMICS was defined as a cigarette in the last 6 months. **Packyears of smoking was calculated** as the number of years of smoking × (cigarettes per day/20) **using standard questionnaire items.** In the MESA Lung Study and SPIROMICS, current smoking was confirmed by urinary cotinine level of >100 ng/mL on the day of CT examination. Urinary cotinine in the MESA Lung Study was measured by immunoassay (Immulite 2000 Nicotine Metabolite Assay, Diagnostic Products Corp., Los Angeles, CA) **and in SPIROMICS by quantitative liquid chromatographic-tandem mass spectrometry (ARUP Laboratories, Salt Lake City, UT).** Estimated glomerular filtration rate (eGFR) was estimated using the four-variable Modification of Diet in Renal Disease equation in the MESA Lung Study.

**Additional Measures**

One of five board-certified radiologists reviewed scans performed in the MESA Lung Study for interstitial lung abnormalities (ILAs), defined as the presence of ground-glass, reticular abnormality, diffuse centrilobular nodularity, honeycombing, traction bronchiectasis, nonemphysematous cysts, or architectural distortion in at least 5% of nondependent portions of the lung (E17).

HAA was defined as the percentage of imaged lung volume having CT attenuation between −600 and −250 Hounsfield units (HU), as previously described (E18).

Spirometry in the MESA Lung Study was conducted in accordance with American Thoracic Society/European Respiratory Society guidelines (E19) on a dry-rolling-sealed spirometer with automated quality checks in real time (Occupational Marketing, Inc., Houston, TX). All spirometry exams were reviewed and graded for quality (E20). Low-quality spirometry was defined as only one acceptable curve; participants with no acceptable curves were excluded. The ICC of both the FEV_1_ and FVC was 0.99.

**Statistical Analysis**

Aptamer levels were natural log transformed to improve normality of distributions. The cross-sectional relationships between percent emphysema (outcome) and aptamers (exposures) were assessed via linear regression models adjusted for height, weight, age, sex, race/ethnicity, PCs of ancestry, educational attainment, smoking status, packyears, urinary cotinine level, eGFR, scanner dose and manufacturer. Beta coefficients were expressed per standard deviation of each aptamer. Height and weight were adjusted for separately, as percent emphysema varies by each independently (E11). Asian participants in SPIROMICS were grouped with American Indian or Alaska Native, Native Hawaiian or other Pacific Islander, and mixed participants given their small sample size. PCs of ancestry in the COPDGene Study were derived within each subgroup of race/ethnicity and thus not used. Urinary cotinine level was not available in the COPDGene Study. eGFR was available only in the MESA Lung Study and adjusted for as renal function can significantly influence circulating plasma protein concentrations (E21). Statistical significance was defined with a false discovery rate (FDR) p-value (q-value) <0.05 using the Benjamini-Hochberg method (E22) and replication required aptamer coefficient estimates to be in the same direction.

To ensure that replicated aptamers were not confounded by restrictive lung disease, a sensitivity analysis excluding participants with pre-bronchodilator restriction on spirometry was conducted. Similarly, to ensure that replicated aptamers were not confounded by interstitial lung disease (ILD), ILAs and HAAs were adjusted for in two additional analyses. Additional sensitivity analyses used deferent parameterizations of smoking variables, were stratified by smoking status, and used a type III analysis of variance (ANOVA) to evaluate for significant interaction terms by smoking status.

Enrichment analyses were performed separately for Gene Ontology (GO) biological processes and Reactome pathways (including protein interactors) using replicated aptamers (E23-E24). Only pathways that achieved FDR-significance and involved at least three proteins were reported.

A least absolute shrinkage and selection operator (LASSO) regression model was conducted in the MESA Lung Study to address collinearity between replicated aptamers, maintaining clinical covariates as unpenalized predictors. Predictive performance was evaluated using ten-fold cross-validated R^2^ for three models including LASSO-selected aptamers alone, unpenalized covariates alone, and a combined model. The incremental R^2^ for each LASSO-selected aptamer was calculated by adding it individually to the covariate-only model.

Analyses were performed using *RStudio* Version 2024.12.0+467 (*R* Foundation, Vienna, Austria) and packages included car (E25), clusterProfiler (E26), data.table (E27), dplyr (E28), enrichplot (E29), glmnet (E30), ggplot2 (E31), ggraph (E32), ggrepel (E33), grid, haven (E34), forcats (E28), igraph (E35), org.Hs.eg.db (E36), patchwork (E37), pheatmap (E38), readxl (E39), sas7bdat (E40), SomaDataIO (E41), stats, tibble (E28), tidygraph (E28), tidyr (E28), tidyverse (E28), viridis (E42), and writexl (E43).

**Supplemental References**

**E1.** Bild DE, Bluemke DA, Burke GL, Detrano R, Diez Roux AV, Folsom AR, et al. Multi-Ethnic Study of Atherosclerosis: Objectives and design. *Am J Epidemiol.*2002 Nov 1;156(9):871–81.

**E2.** Rodriguez J, Jiang R, Johnson WC, MacKenzie BA, Smith LJ, Barr RG. The association of pipe and cigar use with cotinine levels, lung function, and airflow obstruction: a cross-sectional study. *Ann Intern Med*. 2010 Feb 16;152(4):201-10.

**E3.** Couper D, LaVange LM, Han M, Barr RG, Bleecker E, Hoffman EA, et al.; SPIROMICS Research Group. Design of the Subpopulations and Intermediate Outcomes in COPD Study (SPIROMICS). *Thorax*. 2014 May;69(5):491-94.

**E4.** Lowe KE, Regan EA, Anzueto A, Austin E, Austin JHM, Beaty TH, et al. COPDGene^®^ 2019: Redefining the Diagnosis of Chronic Obstructive Pulmonary Disease. *Chronic Obstr Pulm Dis*. 2019 Nov;6(5):384-99.

**E5.** Gold L, Ayers D, Bertino J, Bock C, Bock A, Brody EN, et al. Aptamer-based multiplexed proteomic technology for biomarker discovery. PLoS One. 2010 Dec 7;5(12):e15004.

**E6.** Gold L, Walker JJ, Wilcox SK, Williams S. Advances in human proteomics at high scale with the SOMAscan proteomics platform. *N Biotechnol.* 2012 Jun 15;29:543-49.

**E7.** Candia J, Daya GN, Tanaka T, Ferrucci L, Walker KA. Assessment of variability in the plasma 7k SomaScan proteomics assay. *Sci Rep.* 2022 Oct 13;12(1):17147.

**E8.** Candia J, Cheung F, Kotliarov Y, Fantoni G, Sellers B, Griesman T, et al. Assessment of Variability in the SOMAscan Assay. *Sci Rep.* 2017 Oct 27;7(1):14248.

**E9.** Dubin RF, Deo R, Ren Y, Lee H, Shou H, Feldman H, et al. Analytical and Biological Variability of a Commercial Modified Aptamer Assay in Plasma Samples of Patients with Chronic Kidney Disease. *J Appl Lab Med.* 2023 May 4;8(3):491-503.

**E10.** Sieren JP, Newell JD Jr, Barr RG, Bleecker ER, Burnette N, Carretta EE, et al.; SPIROMICS Research Group. SPIROMICS Protocol for Multicenter Quantitative Computed Tomography to Phenotype the Lungs. *Am J Respir Crit Care Med*. 2016 Oct 1;194(7):794-806.

**E11.** Hoffman EA, Ahmed FS, Baumhauer H, Budoff M, Carr JJ, Kronmal R, et al. Variation in the percent of emphysema-like lung in a healthy, nonsmoking multiethnic sample. The MESA lung study. *Ann Am Thorac Soc*. 2014 Jul;11(6):898-907.

**E12.** Regan EA, Hokanson JE, Murphy JR, Make B, Lynch DA, Beaty TH, et al. Genetic epidemiology of COPD (COPDGene) study design. *COPD*. 2010 Feb;7(1):32-43.

**E13.** Gevenois PA, De Vuyst P, de Maertelaer V, Zanen J, Jacobovitz D, Cosio MG, et al. Comparison of computed density and microscopic morphometry in pulmonary emphysema. *Am J Respir Crit Care Med*. 1996 Jul;154(1):187-92.

**E14.** Gevenois PA, de Maertelaer V, De Vuyst P, Zanen J, Yernault JC. Comparison of computed density and macroscopic morphometry in pulmonary emphysema. *Am J Respir Crit Care Med.* 1995 Aug;152(2):653–57.

**E15.** Motahari A, Barr RG, Han MK, Anderson WH, Barjaktarevic I, Bleecker ER, et al.; SPIROMICS Group. Repeatability of Pulmonary Quantitative Computed Tomography Measurements in Chronic Obstructive Pulmonary Disease. *Am J Respir Crit Care Med*. 2023 Sep 15;208(6):657-65.

**E16.** Powell R, Davidson D, Divers J, Manichaikul A, Carr JJ, Detrano R, et al. Genetic ancestry and the relationship of cigarette smoking to lung function and percent emphysema in four race/ethnic groups: a cross-sectional study. *Thorax*. 2013 Jul;68(7):634-42.

**E17.** Washko GR, Hunninghake GM, Fernandez IE, Nishino M, Okajima Y, Yamashiro T, et al.; COPDGene Investigators. Lung volumes and emphysema in smokers with interstitial lung abnormalities. *N Engl J Med*. 2011 Mar 10;364(10):897-906.

**E18.** Lederer DJ, Enright PL, Kawut SM, Hoffman EA, Hunninghake G, van Beek EJ, et al. Cigarette smoking is associated with subclinical parenchymal lung disease: the Multi-Ethnic Study of Atherosclerosis (MESA)-lung study. *Am J Respir Crit Care Med*. 2009 Sep 1;180(5):407-14.

**E19.** Miller MR, Hankinson J, Brusasco V, Burgos F, Casaburi R, Coates A, et al. Standardisation of spirometry. *Eur Respir J.*2005 Aug;26(2):319–38.

**E20.** Hankinson JL, Kawut SM, Shahar E, Smith LJ, Stukovsky KH, Barr RG. Performance of American Thoracic Society-recommended spirometry reference values in a multiethnic sample of adults: the multi-ethnic study of atherosclerosis (MESA) lung study. *Chest*. 2010 Jan;137(1):138-45.

**E21.** Yang J, Brody EN, Murthy AC, Mehler RE, Weiss SJ, DeLisle RK, et al. Impact of Kidney Function on the Blood Proteome and on Protein Cardiovascular Risk Biomarkers in Patients With Stable Coronary Heart Disease. *J Am Heart Assoc*. 2020 Aug 4;9(15):e016463.

**E22.** Benjamini Y, Hochberg Y. Controlling the False Discovery Rate: A Practical and Powerful Approach to Multiple Testing. *Journal of the Royal Statistical Society: Series B (Methodological).* 1995;57:289-300.

**E23.** Ashburner M, Ball CA, Blake JA, Botstein D, Butler H, Cherry JM, et al. Gene ontology: tool for the unification of biology. The Gene Ontology Consortium. *Nat Genet*. 2000 May;25(1):25-9.

**E24.** Fabregat A, Sidiropoulos K, Viteri G, Forner O, Marin-Garcia P, Arnau V, et al. Reactome pathway analysis: a high-performance in-memory approach. *BMC bioinformatics*. 2017 Mar;18(1) 142.

**E25.** Fox J, Weisberg S. An R Companion to Applied Regression, Third Edition. *Sage*. 2019.

**E26.** Yu G, Wang LG, Han Y, He QY. clusterProfiler: an R package for comparing biological themes among gene clusters. *OMICS*. 2012 May;16(5):284-7.

**E27.** Barrett T, Dowle M, Srinivasan A, Gorecki J, Chirico M, Hocking T, et al. data.table: Extension of 'data.frame'. *R package version 1.17.99.* 2025.

**E28.** Wickam H, Averick M, Bryan J, Chang W, McGowan LD, Francois R, et al. Welcome to the Tidyverse. *Journal of Open Source Software*. 2019;4(43):1686.

**E29.** Yu G. Enrichplot: visualization of functional enrichment result. *R package version 1.28.2.* 2021;1*.*

**E30.** Friedman J, Hastie T, Tibshirani R. Regularization Paths for Generalized Linear Models via Coordinate Descent. *J Stat Softw*. 2010;33(1):1-22.

**E31.** Wickham H. ggplot2: Elegant Graphics for Data Analysis. *Springer-Verlag New York*. 2016.

**E32.** Pedersen TL. An Implementation of Grammar of Graphics for Graphs and Networks. *R package version 2.2.1.* 2020.

**E33.** Slowikowski K, Schep A, Hughes S, Dang TK, Lukauskas S, Irisson JO, et al. Automatically Position Non-Overlapping Text Labels with ‘ggplot2’. *R package version 0.9.6.* 2024.

**E34.** Wickham H, Miller E, Smith D. haven: Import and Export ‘SPSS’, ‘Stata’ and ‘SAS’ Files. *R package version 2.5.5.* 2025.

**E35.** Csardi G, Nepusz T. The Igraph Software Package for Complex Network Research. *InterJournal Complex Systems*. 2006;1695.

**E36.** Carlson M, Falcon S, Pages H, Li N. Genome wide annotation for Human. *R package version 3.21.0.* 2021.

**E37.** Pederson TL. The Composer of Plots. *R package version 1.3.0.* 2020.

**E38.** Kolde R. Pretty Heatmaps. *R package version 1.0.13.* 2019.

**E39.** Wickham H, Bryan J, Kalicinski M, Valery K, Leitienne C, Colbert B, et al. Read Excel Files. *R package version 1.4.5.* 2021.

**E40.** Shotwell M, Cummins C. sas7bdat Reverse Engineering Documentation. *R package version 0.8.* 2024.

**E41.** Field S, Scheidel C, Standard BioTools, Inc. SomaDataIO: Input/Output “SomaScan” Data. *R package version 6.3.0.* 2025.

**E42.** Garnier S, Ross N, Rudis R, Camargo AP, Sciaini M, Scherer C. viridisLite: Colorblind-Friendly Color Maps (Lite Version). *R package version 0.4.2.* 2023.

**E43.** Ooms J. writexl: Data Frames to Excel ‘xlsx’ Format. *R package version 1.5.4.* 2020.

| **Table E2:** Natural log transformed mean and standard deviation (SD) of replicated aptamers associated with percent emphysema. | | | | | | | |
| --- | --- | --- | --- | --- | --- | --- | --- |
|  | | **MESA Lung** | | **SPIROMICS** | | **COPDGene** | |
| **Protein Name** | **Gene ID** | **Mean** | **SD** | **Mean** | **SD** | **Mean** | **SD** |
| Protein FAM177A1 | **FAM177A1** | 8.1 | 0.25 | 8.1 | 0.23 | 8.0 | 0.27 |
| Syntenin-2 | **SDCBP2** | 6.1 | 0.30 | 6.2 | 0.32 | 5.9 | 0.34 |
| Peroxisomal NADH pyrophosphatase NUDT12 | **NUDT12** | 7.4 | 0.36 | 7.4 | 0.29 | 7.3 | 0.30 |
| Ubiquitin carboxyl-terminal hydrolase 25 | **USP25** | 6.7 | 0.34 | 6.6 | 0.29 | 6.7 | 0.29 |
| Serine/threonine-protein kinase MRCK beta | **CDC42BPB** | 7.6 | 0.35 | 7.6 | 0.26 | 7.9 | 0.39 |
| Uncharacterized protein C20orf173 | **C20orf173** | 6.8 | 0.28 | 6.8 | 0.19 | 6.9 | 0.22 |
| Apoptosis Regulator Bcl-2 | **BCL2** | 6.8 | 0.42 | 6.9 | 0.31 | 7.0 | 0.41 |
| Leukocyte elastase inhibitor | **SERPINB1** | 8.6 | 0.38 | 8.6 | 0.38 | 8.7 | 0.48 |
| EGF-like repeat and discoidin I-like domain-containing protein 3 | **EDIL3** | 7.4 | 0.27 | 6.8 | 0.42 | 7.4 | 0.46 |
| Polypeptide N-acetylgalactosaminyltransferase 16 | **GALNT16** | 8.4 | 0.24 | 8.0 | 0.33 | 7.8 | 0.34 |
| Advanced glycosylation end product-specific receptor, soluble | **AGER** | 8.0 | 0.62 | 7.6 | 0.62 | 7.9 | 0.69 |
| Fibroleukin | **FGL2** | 7.1 | 0.21 | 7.2 | 0.19 | 7.4 | 0.25 |
| Protein S100-A12 | **S100A12** | 7.9 | 0.41 | 7.8 | 0.32 | 8.0 | 0.42 |
| IQ domain-containing protein F3 | **IQCF3** | 7.2 | 0.32 | 7.1 | 0.23 | 7.0 | 0.33 |
| Protein S100-A9 | **S100A9** | 8.3 | 0.45 | 8.2 | 0.46 | 8.3 | 0.56 |
| EKC/KEOPS complex subunit TPRKB | **TPRKB** | 6.9 | 0.22 | 6.9 | 0.17 | 7.0 | 0.19 |
| Interleukin-1 receptor antagonist protein | **IL1RN** | 7.3 | 0.36 | 7.2 | 0.24 | 7.8 | 0.33 |
| AMMECR1-like protein | **AMMECR1L** | 5.9 | 0.42 | 6.0 | 0.34 | 6.0 | 0.39 |
| Polypeptide N-acetylgalactosaminyltransferase 11 | **GALNT11** | 7.4 | 0.26 | 7.3 | 0.15 | 7.4 | 0.17 |
| Kremen protein 2 | **KREMEN2** | 8.2 | 0.41 | 8.3 | 0.28 | 8.5 | 0.36 |
| Calponin-1 | **CNN1** | 8.0 | 0.32 | 8.0 | 0.36 | 8.0 | 0.32 |
| Coagulation factor IX | **F9** | 10.0 | 0.19 | 10.0 | 0.16 | 9.6 | 0.18 |
| WAP four-disulfide core domain protein 1 | **WFDC1** | 8.9 | 0.29 | 8.7 | 0.27 | 8.8 | 0.30 |
| Coagulation Factor IXab | **F9** | 9.5 | 0.20 | 9.4 | 0.22 | 9.0 | 0.21 |
| Zona pellucida-binding protein 1 | **ZPBP** | 8.2 | 0.23 | 8.0 | 0.12 | 8.0 | 0.12 |
| Teneurin-2 | **TENM2** | 7.8 | 0.28 | 7.7 | 0.14 | 7.9 | 0.19 |
| Apolipoprotein D | **APOD** | 8.1 | 0.28 | 8.1 | 0.27 | 8.3 | 0.31 |
| Roundabout homolog 2 | **ROBO2** | 7.3 | 0.18 | 7.1 | 0.16 | 7.3 | 0.16 |
| Procollagen-lysine,2-oxoglutarate 5-dioxygenase 3 | **PLOD3** | 7.5 | 0.30 | 7.6 | 0.25 | 7.7 | 0.31 |
| Heterogeneous nuclear ribonucleoprotein D0 | **HNRNPD** | 7.9 | 0.49 | 8.1 | 0.39 | 8.3 | 0.44 |
| Kynureninase | **KYNU** | 7.6 | 0.30 | 7.5 | 0.20 | 7.6 | 0.25 |
| Adiponectin | **ADIPOQ** | 8.2 | 0.47 | 8.2 | 0.47 | 8.1 | 0.48 |
| High mobility group protein B1 | **HMGB1** | 8.8 | 0.40 | 8.9 | 0.29 | 9.1 | 0.32 |
| Integral membrane protein 2A | **ITM2A** | 7.6 | 0.30 | 7.5 | 0.18 | 7.6 | 0.19 |
| Activator of 90 kDa heat shock protein | **AHSA1** | 8.5 | 0.41 | 8.7 | 0.33 | 8.7 | 0.40 |

| **Table E3:** Sensitivity analysis of replicated aptamers associated with percent emphysema in the MESA Lung Study in participants without restriction on spirometry and with adjustment for interstitial lung abnormalities (ILAs) and high attenuation areas (HAAs). | | | | | | | |
| --- | --- | --- | --- | --- | --- | --- | --- |
|  | | **Participants without restriction on spirometry**  **(n = 1,999)** | | **Adjusting for ILAs**  **(n = 2,075)** | | **Adjusting for HAAs**  **(n = 2,442)** | |
| **Protein Name** | **Gene ID** | **q-value** | **β Coefficient*** | **q-value** | **β Coefficient*** | **q-value** | **β Coefficient*** |
| Protein FAM177A1 | **FAM177A1** | 0.004 | 4.65 | 0.008 | 3.73 | 0.003 | 3.66 |
| Syntenin-2 | **SDCBP2** | 0.007 | 2.80 | 0.024 | 2.00 | 0.001 | 2.67 |
| Peroxisomal NADH pyrophosphatase NUDT12 | **NUDT12** | 0.011 | 1.78 | 0.004 | 1.89 | 0.005 | 1.55 |
| Ubiquitin carboxyl-terminal hydrolase 25 | **USP25** | 0.003 | 2.39 | 0.003 | 2.35 | 0.002 | 1.98 |
| Serine/threonine-protein kinase MRCK beta | **CDC42BPB** | 0.011 | 1.91 | 0.008 | 1.79 | 0.003 | 1.78 |
| Uncharacterized protein C20orf173 | **C20orf173** | 0.012 | 3.01 | 0.007 | 3.02 | 0.006 | 2.62 |
| Apoptosis Regulator Bcl-2 | **BCL2** | 0.013 | 1.30 | 0.011 | 1.18 | 0.004 | 1.23 |
| Leukocyte elastase inhibitor | **SERPINB1** | 0.012 | 1.60 | 0.023 | 1.25 | 0.001 | 1.70 |
| EGF-like repeat and discoidin I-like domain-containing protein 3 | **EDIL3** | 0.073 | -2.27 | 0.022 | -2.48 | 0.047 | -1.91 |
| Polypeptide N-acetylgalactosaminyltransferase 16 | **GALNT16** | 0.027 | -3.62 | 0.028 | -3.26 | 0.059 | -2.38 |
| Advanced glycosylation end product-specific receptor, soluble | **AGER** | 0.035 | -0.53 | 0.063 | -0.42 | 0.022 | -0.46 |
| Fibroleukin | **FGL2** | 0.018 | 4.73 | 0.009 | 4.81 | 0.006 | 4.43 |
| Protein S100-A12 | **S100A12** | 0.038 | 1.10 | 0.035 | 1.02 | 0.003 | 1.35 |
| IQ domain-containing protein F3 | **IQCF3** | 0.033 | 1.82 | 0.003 | 2.39 | 0.005 | 1.91 |
| Protein S100-A9 | **S100A9** | 0.029 | 0.93 | 0.019 | 0.91 | 0.004 | 1.05 |
| EKC/KEOPS complex subunit TPRKB | **TPRKB** | 0.024 | 3.93 | 0.013 | 4.21 | 0.007 | 4.07 |
| Interleukin-1 receptor antagonist protein | **IL1RN** | 0.020 | 1.85 | 0.006 | 2.01 | 0.002 | 1.95 |
| AMMECR1-like protein | **AMMECR1L** | 0.016 | 1.27 | 0.008 | 1.23 | 0.005 | 1.14 |
| Polypeptide N-acetylgalactosaminyltransferase 11 | **GALNT11** | 0.033 | 2.72 | 0.019 | 2.83 | 0.009 | 2.84 |
| Kremen protein 2 | **KREMEN2** | 0.029 | 1.12 | 0.019 | 1.13 | 0.016 | 1.04 |
| Calponin-1 | **CNN1** | 0.026 | 1.98 | 0.022 | 1.82 | 0.005 | 2.02 |
| Coagulation factor IX | **F9** | 0.024 | 5.76 | 0.016 | 5.72 | 0.018 | 4.70 |
| WAP four-disulfide core domain protein 1 | **WFDC1** | 0.117 | -1.82 | 0.111 | -1.73 | 0.045 | -1.96 |
| Coagulation Factor IXab | **F9** | 0.027 | 5.13 | 0.018 | 4.99 | 0.020 | 4.19 |
| Zona pellucida-binding protein 1 | **ZPBP** | 0.041 | 3.46 | 0.017 | 3.75 | 0.015 | 3.40 |
| Teneurin-2 | **TENM2** | 0.037 | 2.20 | 0.020 | 2.27 | 0.022 | 2.00 |
| Apolipoprotein D | **APOD** | 0.028 | 2.31 | 0.013 | 2.46 | 0.013 | 2.17 |
| Roundabout homolog 2 | **ROBO2** | 0.065 | -5.48 | 0.044 | -5.26 | 0.162 | -3.13 |
| Procollagen-lysine,2-oxoglutarate 5-dioxygenase 3 | **PLOD3** | 0.035 | 2.04 | 0.013 | 2.33 | 0.016 | 1.95 |
| Heterogeneous nuclear ribonucleoprotein D0 | **HNRNPD** | 0.028 | 0.78 | 0.020 | 0.76 | 0.007 | 0.84 |
| Kynureninase | **KYNU** | 0.041 | 2.02 | 0.038 | 1.89 | 0.009 | 2.18 |
| Adiponectin | **ADIPOQ** | 0.063 | 0.82 | 0.031 | 0.87 | 0.023 | 0.816 |
| High mobility group protein B1 | **HMGB1** | 0.045 | 1.04 | 0.030 | 1.06 | 0.015 | 1.09 |
| Integral membrane protein 2A | **ITM2A** | 0.028 | 2.06 | 0.019 | 2.08 | 0.016 | 1.82 |
| Activator of 90 kDa heat shock protein | **AHSA1** | 0.029 | 1.11 | 0.020 | 1.08 | 0.024 | 0.93 |

*For model 1 in which participants without restriction on spirometry are selected, the β Coefficient represents the multivariable difference in percent emphysema per natural log-scale standard deviation (SD) of each aptamer adjusted for age, height, weight, sex, race/ethnicity, PCs of ancestry, educational attainment, smoking status, packyears, urinary cotinine level, eGFR, and CT scanner dose and manufacturer. For model 2 in which participants have valid measures of ILAs, the β Coefficient represents the multivariable difference in percent emphysema per natural log-scale standard deviation (SD) of each aptamer adjusted for the same original covariates in addition to ILAs. For model 3 in which participants have valid measures of HAAs, the β Coefficient represents the multivariable difference in percent emphysema per natural log-scale standard deviation (SD) of each aptamer adjusted for the same original covariates in addition to HAAs.

| **Table E4:** Sensitivity analysis of replicated aptamers associated with percent emphysema in the MESA Lung Study with alternative parameterization of smoking history. | | | |
| --- | --- | --- | --- |
|  |  | **MESA Lung** | |
| **Protein Name** | **Gene ID** | **q-value** | **β Coefficient*** |
| Protein FAM177A1 | **FAM177A1** | 0.005 | 4.06 |
| Syntenin-2 | **SDCBP2** | 0.012 | 2.31 |
| Peroxisomal NADH pyrophosphatase NUDT12 | **NUDT12** | 0.012 | 1.62 |
| Ubiquitin carboxyl-terminal hydrolase 25 | **USP25** | 0.014 | 1.75 |
| Serine/threonine-protein kinase MRCK beta | **CDC42BPB** | 0.014 | 1.72 |
| Uncharacterized protein C20orf173 | **C20orf173** | 0.014 | 2.64 |
| Apoptosis Regulator Bcl-2 | **BCL2** | 0.015 | 1.17 |
| Leukocyte elastase inhibitor | **SERPINB1** | 0.015 | 1.42 |
| EGF-like repeat and discoidin I-like domain-containing protein 3 | **EDIL3** | 0.015 | -2.76 |
| Polypeptide N-acetylgalactosaminyltransferase 16 | **GALNT16** | 0.024 | -3.30 |
| Advanced glycosylation end product-specific receptor, soluble | **AGER** | 0.026 | -0.52 |
| Fibroleukin | **FGL2** | 0.030 | 3.97 |
| Protein S100-A12 | **S100A12** | 0.029 | 1.09 |
| IQ domain-containing protein F3 | **IQCF3** | 0.030 | 1.65 |
| Protein S100-A9 | **S100A9** | 0.032 | 0.86 |
| EKC/KEOPS complex subunit TPRKB | **TPRKB** | 0.034 | 3.56 |
| Interleukin-1 receptor antagonist protein | **IL1RN** | 0.033 | 1.44 |
| AMMECR1-like protein | **AMMECR1L** | 0.033 | 0.97 |
| Polypeptide N-acetylgalactosaminyltransferase 11 | **GALNT11** | 0.036 | 2.56 |
| Kremen protein 2 | **KREMEN2** | 0.037 | 1.02 |
| Calponin-1 | **CNN1** | 0.037 | 1.70 |
| Coagulation factor IX | **F9** | 0.043 | 4.51 |
| WAP four-disulfide core domain protein 1 | **WFDC1** | 0.036 | -2.36 |
| Coagulation Factor IXab | **F9** | 0.048 | 3.99 |
| Zona pellucida-binding protein 1 | **ZPBP** | 0.046 | 3.12 |
| Teneurin-2 | **TENM2** | 0.047 | 1.98 |
| Apolipoprotein D | **APOD** | 0.048 | 1.93 |
| Roundabout homolog 2 | **ROBO2** | 0.047 | -5.27 |
| Procollagen-lysine,2-oxoglutarate 5-dioxygenase 3 | **PLOD3** | 0.048 | 1.78 |
| Heterogeneous nuclear ribonucleoprotein D0 | **HNRNPD** | 0.047 | 0.66 |
| Kynureninase | **KYNU** | 0.051 | 1.77 |
| Adiponectin | **ADIPOQ** | 0.045 | 0.81 |
| High mobility group protein B1 | **HMGB1** | 0.049 | 0.98 |
| Integral membrane protein 2A | **ITM2A** | 0.052 | 1.64 |
| Activator of 90 kDa heat shock protein | **AHSA1** | 0.054 | 0.89 |

*Multivariable difference in percent emphysema per natural log-scale standard deviation (SD) of each aptamer adjusted for age, height, weight, sex, race/ethnicity, PCs of ancestry, educational attainment, packyears, urinary cotinine level, eGFR, and CT scanner dose and manufacturer.

**Figure E1:** Replicated aptamers associated with percent emphysema stratified by smoking status in the MESA Lung Study.


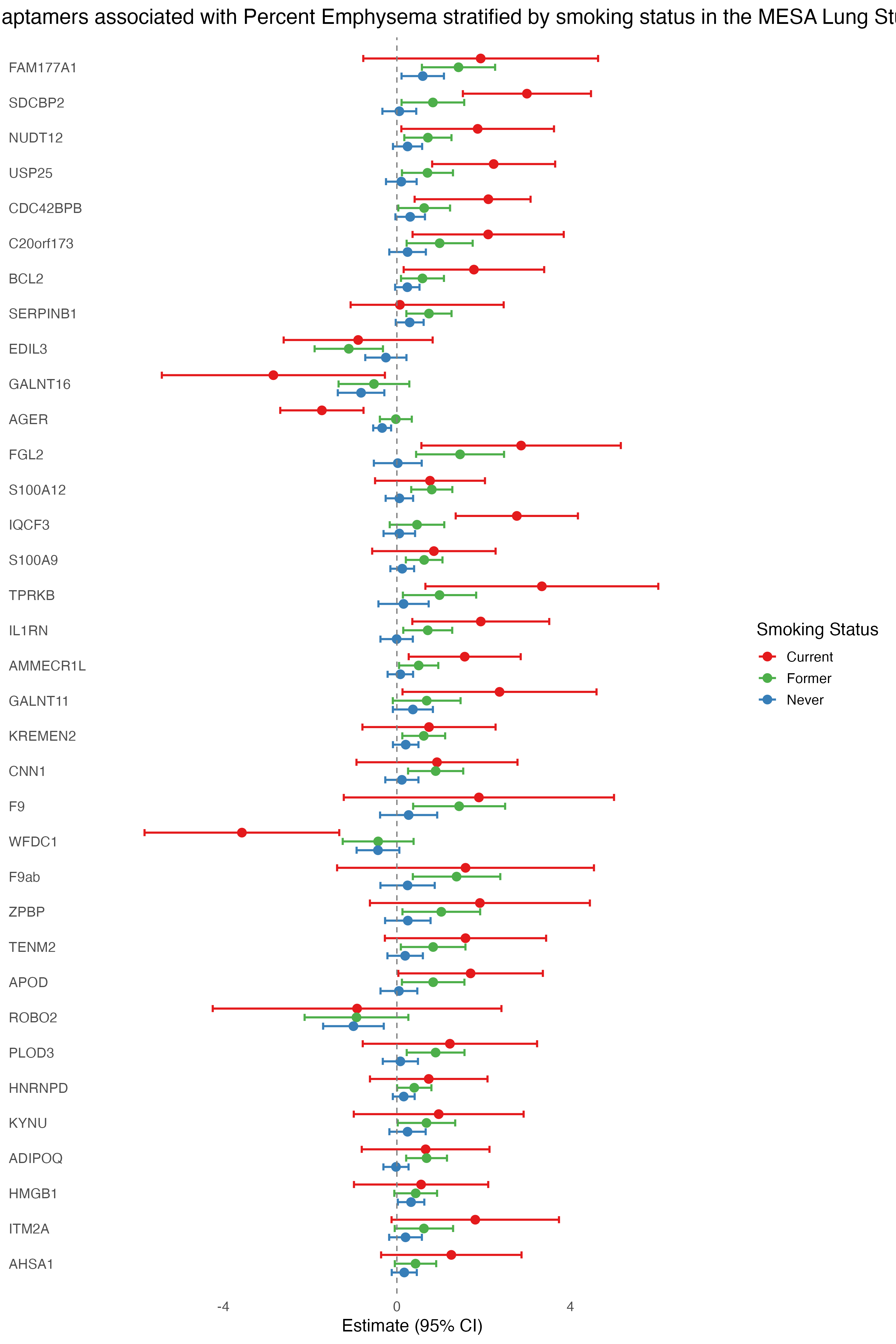

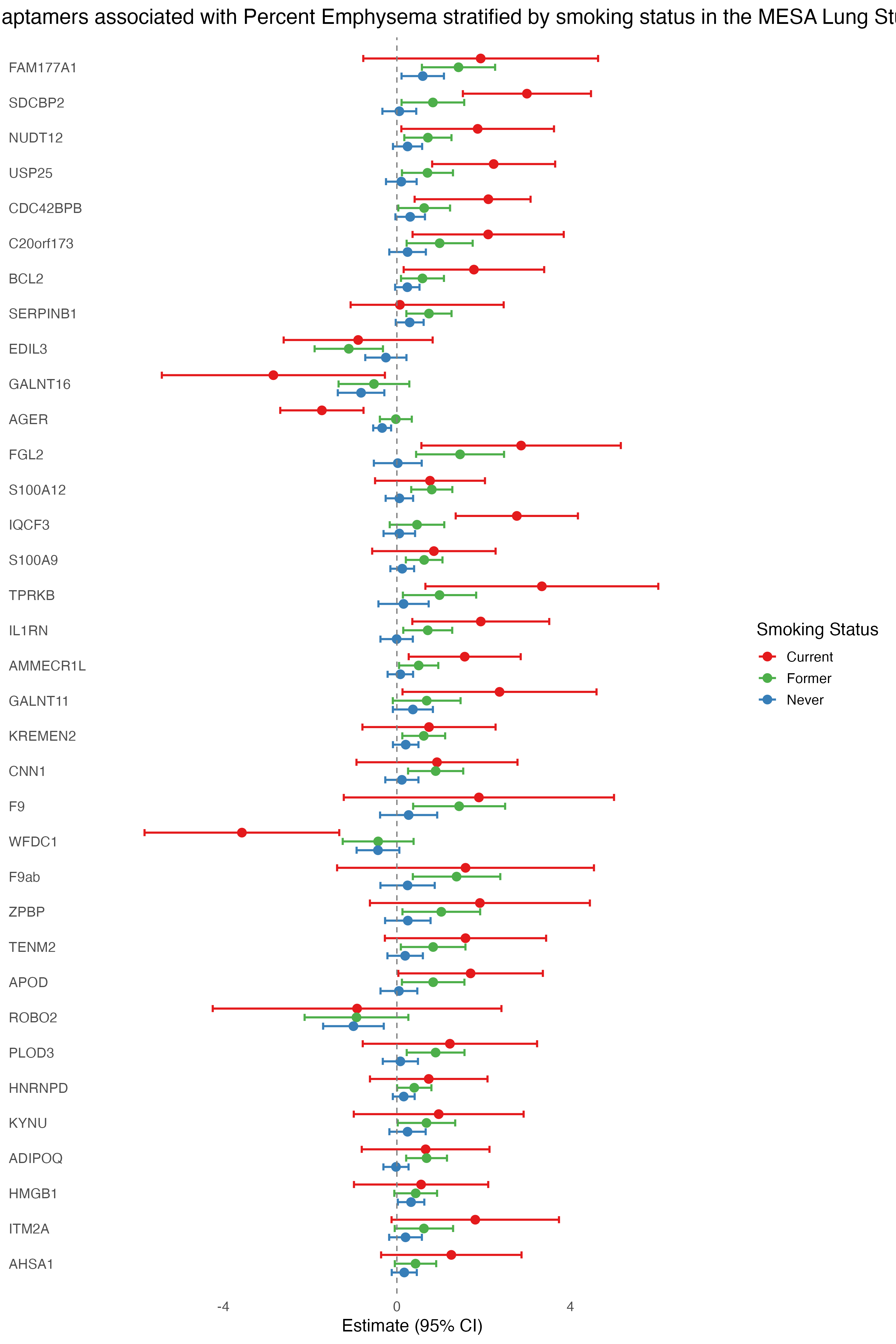


*

*

*

*

*

*

*

*

*Significant interaction terms by smoking status after type III analysis of variance (ANOVA) included syntenin-2 (q<0.001), ubiquitin carboxyl-terminal hydrolase 25 (q=0.014), soluble advanced glycosylation end product-specific receptor (q=0.011), fibroleukin (q=0.030), IQ domain-containing protein F3 (q<0.001), EKC/KEOPS complex subunit TPRKB (q=0.030), AMMECR1-like protein (q=0.028), and adiponectin (q=0.20).
